# Characterising anticipatory postural adjustments in turning a comparison between older adults and people with Parkinson’s disease

**DOI:** 10.1038/s41598-025-33425-5

**Published:** 2025-12-22

**Authors:** Yuri Russo, Phaedra Leveridge, Jiaxi Ye, Zijing Wang, Alice Nieuwboer, Sarah E. Lamb, Elmar Kal, Meriel Norris, Mark Wilson, William R. Young

**Affiliations:** 1 Department of Public Health and Sport Sciences, University of Exeter, Exeter, EX1 2LU UK; 2 Department of Rehabilitation Sciences, Katholieke Universiteit Leuven, Leuven, Belgium; 3 Department of Health Sciences, Brunel University of London, London, UK

**Keywords:** Turn, Biomechanics, APA, Step, Posture, Parkinson’s disease, Parkinson's disease, Biomedical engineering

## Abstract

Anticipatory postural adjustments (APAs) are crucial for maintaining postural stability during voluntary movements such as gait initiation. While APAs have been extensively studied in forward stepping, little is known about their characteristics during turning initiation. This study aimed to identify the characteristics of (i) APAs and subsequent first steps during turning in older adults (OA) and (ii) compare them to people with Parkinson’s disease (pwP). Twenty-two pwP (tested on medication) and 17 OA performed self-paced 360˚ turns which were embedded in a complex walking task. APAs and first step characteristics were recorded using motion capture and force plate data. For pwP, APAs in turning (unloading phase) were found to be primarily mediolateral, and of significantly reduced amplitude (median = 2.5, 95% CI[0.0053; 0.0089]) in comparison to OA (median = 5.0, 95% CI[0.0073; 0.0181]). Unlike OA there was no significant association between APAs and step characteristics. These findings suggest that APAs during turning are significantly impaired in pwP, even when tested ON medication, and that this impairment may contribute to the turning difficulties often experienced by this population. Overall, these results have potential implications for clinical assessments and rehabilitation interventions, emphasising the need to tailor strategies to address turning challenges pwP face in their daily life.

## Introduction

Voluntary stepping movements are often preceded by Anticipatory Postural Adjustments (APAs). These preparatory adjustments rely on specific motor programmes generated by the central nervous system before the intended movement^1,2^ to promote postural stability during the initiated step^3,4^. APAs are modulated according to environmental demands and conditions (e.g., time constraints, emotional state, base of support^5,6^). In forward gait initiation, APAs shift the centre of mass forwards and towards the standing limb i.e., unloading the stepping leg and preparing the body to accelerate forward^4^. APAs can be divided into two phases: imbalance and unloading^7^. The first promotes unloading of the stepping leg while accelerating the centre of mass forward, and the second represents the weight-shift associated with preparation for mono-pedal stance and heel-off.

APAs preceding gait initiation are shown to be stable in healthy people^8^ and are not significantly influenced by healthy ageing^9,10^. However, APAs are often studied in clinical contexts as they significantly influence first-step characteristics and walking performance^5,11,12^. Established evidence shows that APAs are impaired in clinical populations^5,13,14^, particularly in people with Parkinson’s (pwP), where defective APAs contribute to difficulties with gait initiation^12,15^. In pwP, falls often occur during attempted postural transitions such as gait initiation and turning^16^), when effective APAs are critical to successful and safe step initiation. Despite their potential clinical relevance^17^, our current understanding of APAs primarily stems from studies on forward step/gait initiation, while non-linear postural transitions (e.g., lateral stepping, initiation of turning) have received little attention^18,19^. This is surprising given that many tasks in daily life involve turning while stepping^20^ and that pwP find it difficult to attempt turning tasks, even in early stages of the disease^21^. In addition, turning behaviour has been shown to be more abnormal in pwP with freezing of gait^22^.

Compared to forward gait initiation, turning is more complex as it requires simultaneous translation and re-orientation of several body segments, as well as posing challenges of postural control. It is therefore more difficult to study given the limitations of conventional biomechanical/clinical laboratories which are often optimised for gait analysis. By using VSimulators laboratory (VSimulators Exeter), we were able to largely overcome these limitations by designing a complex walking task that broadly resembles common real-world demands without sacrificing the use of gold-standard technology (i.e., motion capture and force plates).

Our research aimed to: i) characterise APAs and properties of the first step during turning in older adults; ii) compare these to pwP; iii) explore whether there are asymmetries in APAs in pwP when turning towards their most versus least affected side. We hypothesised that: i) APAs in turning would manifest primarily in the mediolateral direction, with small APAs in the anteroposterior direction; ii) pwP would show longer and smaller APAs compared to older adults; iii) we did not have a specific hypothesis regarding potential asymmetries in APAs between the most and least affected sides in pwP. Furthermore, since balance ability may influence turning strategy and performance, we also explored whether individual differences in balance ability were associated with turning characteristics.

## Methods

### Study design

This cross-sectional study characterised APAs and first-step characteristics in turning and compared these outcomes between older adults and pwP. All participants were tested at the VSimulators laboratory (VSimulators Exeter), University of Exeter, between March 2022 and October 2022. All participants with Parkinson’s disease (PD) were tested while in their ON-medication state (~ 60 min after taking their regular dopaminergic medication). If necessary, participants were allowed to re-administer their medication to ensure they remained in their ON state throughout the testing.

### Participants

Thirty-six pwP and twenty-four older adults were recruited through the Parkinson’s UK research network and the local community. Participants were eligible if they: i) were over 55 years old; ii) were able to walk unsupported for at least one minute. An additional inclusion criterion for the PD group was an existing diagnosis of idiopathic PD (UK Brain Bank Criteria). Exclusion criteria for all participants were: i) moderate cognitive impairment (Montreal Cognitive Assessment, MOCA, score < 21^23^); ii) impaired normal or corrected-to-normal vision (Snellen Visual Acuity < 12/18); and iii) any injury or disorder (other than PD) that might affect balance or walking. PwP were asked to answer the first question of the New Freezing of Gait Questionnaire (NFOG-Q,^24^) to self-identify whether they experience freezing. All participants provided written informed consent prior to participating in the study. The study was approved by the local institutional review board of the University of Exeter (21-12-08-B- 02, Department of Public Health & Sport Sciences), and all experimental procedures were performed in accordance with relevant guidelines and regulations.

### Procedure

**Fig. 1:**
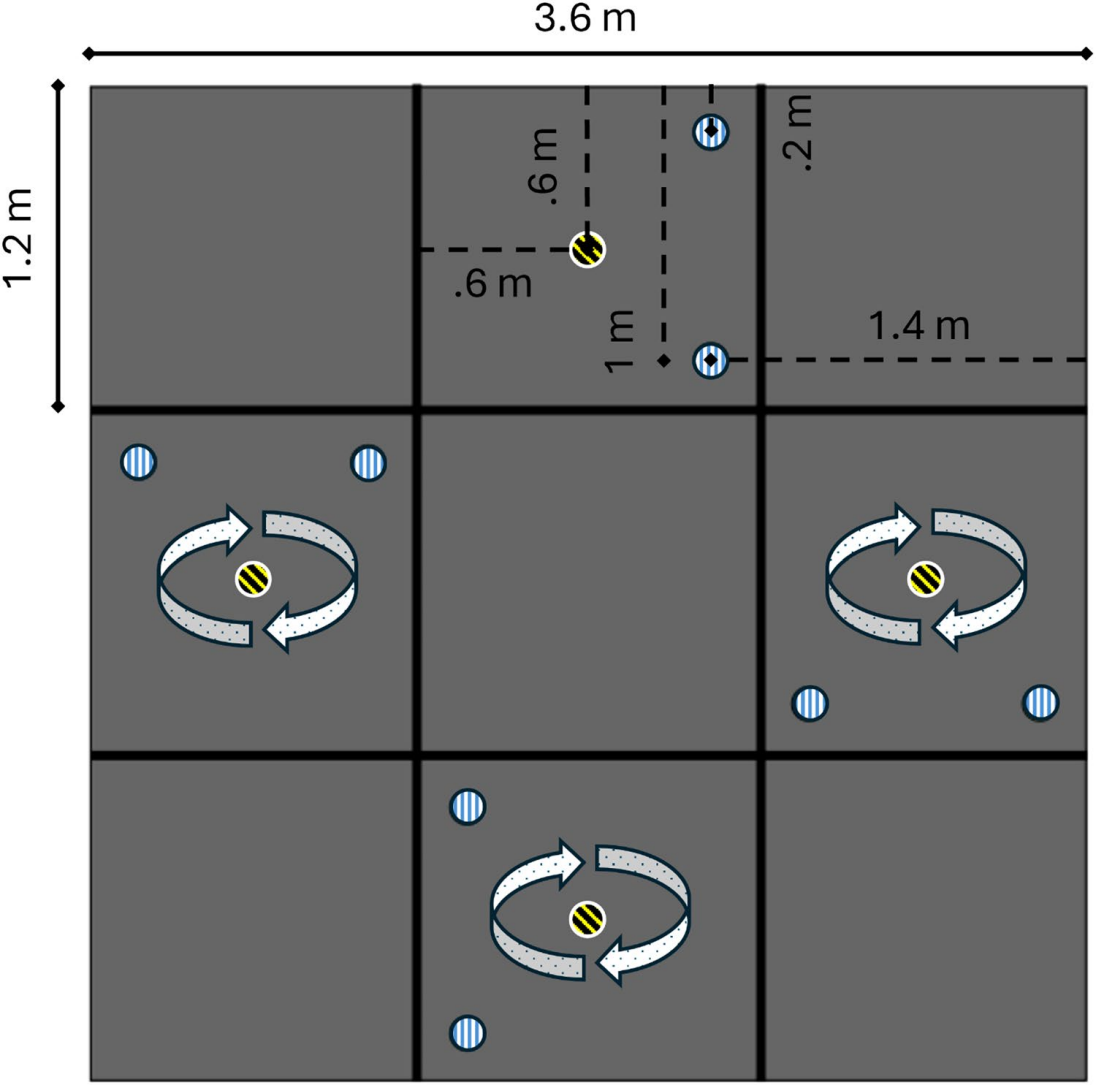
Graphical representation of the walking task. Grey squares represent the positions of the force plates. Vertical dashed circles indicate the locations of the cones, while the black-and-yellow circle with oblique dashes marks the target where participants stopped and performed a 360° turn. Circular dotted arrows show the points where participants executed turns. Black dashed lines illustrate the relative positions of the targets within the instrumented floor, whereas black solid lines show the overall dimensions of the instrumented floor (outer perimeter).

After providing written informed consent, pwP were assessed by a trained examiner (YR or WY) using the Mini-BESTest^25^ and the Motor Section (Part III) of the MDS-UPDRS which consists of 23 items evaluating bradykinesia, rigidity, tremor, posture and gait^26^. They then underwent a walking test, designed to capture full body motion during turning and other elements of gait. The walking task was arranged in a square pattern with an external width/length of 3.6 m × 3.6 m. At regular intervals on each side of the square, a visual target of 15 cm diameter was taped to the floor (see Fig. 1). The target was black and yellow and had a high visual contrast with the floor (light grey). Following an initial standing period, participants were instructed to walk forward and stop on the visual target. They were asked to pause for a few seconds, before performing a full 360˚ turn towards a self-selected direction (i.e., clockwise or counterclockwise) at a self-selected speed. On completing the turn, participants were asked to stop for a couple of seconds before walking through two cones (positioned ~ 0.2 m and ~ 1 m from the outer lateral perimeter of the walkway and ~ 1.4 m from the outer perimeter in the direction of the walking, to guide the navigation of the space), perform a 90˚turn, and continue walking until reaching the next visual target. To have more naturalistic turning and avoid influencing participants’ natural behaviour, no feedback or additional instructions were given during the walking task. All participants completed the task (i.e., walking along the square pattern) both in the clockwise and counterclockwise directions. Each participant completed at least six 360˚ turns in each walking direction. Before testing, an experimenter (YR or WY) provided the task instructions both verbally and through demonstration. Participants were then asked to familiarise with the environment and the task by going through the testing protocol a few times.

### Instruments

Kinematic data were collected using a motion capture system with 19 cameras (100 Hz, Prime^x^ 13, Optitrack, USA). Participants were fitted with 6 retro-reflective markers positioned on 3 anatomical landmarks of each foot (heel, lateral malleolus, and head of the first metatarsus). Kinetic data were collected using 9 force plates (1.2 m × 1.2 m each, 1000 Hz, AMTI, USA), arranged in a square pattern, covering an area of ~ 13 m^2^. The experimental task was designed to maximise participants’ navigation of the space, while ensuring that all walking occurred on the force plates array (3 × 3). A video camera (25 Hz, HDR-CX405, Sony) was used to record the trials to allow the identification of turns affected by freezing of gait^27^.

### Data analysis and outcome variables

Video, kinematic and kinetic data relating to all turns were visually inspected and analysed semi-automatically. Data were processed using customised scripts and functions written in Matlab (R2023a, MathWorks, Natick, MA, USA). Kinematic and kinetic data were low pass filtered using a fourth order Butterworth filter with a cut-off frequency of 15 Hz^28^. The filtered kinematic data were used to identify the time windows of the turns based on the physical position of the turn target within the motion capture reference system. This followed a two-step approach:

(i) The time between the stop and the beginning of the APA (defined as in^8^) was estimated. If this time was shorter than 3 s, the trial was excluded from further analysis to avoid centre of pressure (CoP) waveforms being affected by the short transition (i.e., absence of quiet stance).
(ii) If the time was longer than 3 s, the turn was deemed suitable for the analysis. A second time window was then extracted, from the moment the participant fully stopped with both feet on the visual target to the completion of the first step of the turn.

Although this approach led to the exclusion of a significant number of turns (59%), it represents a conservative method to ensure data quality. Additionally, allowing participants to choose their initial turn direction and perform the task at a self-selected pace enhanced the generalisability of the findings to real-world scenarios.

Moments and force components were used to calculate the CoP coordinates. The CoP trajectories were extracted based on the time windows described above. The reference system used to represent the CoP trajectory during the time window was rotated following the orientation of the base of support (BoS) and then adjusted depending on the leading limb and the turning direction, allowing for the comparison of the CoP waveforms (see^8^). To reliably allow the identification of the onset of APAs, a threshold value was set for each turn window as three standard deviations above the baseline value during a 1 s quiet standing period (taken from the time window immediately preceding the turn, see above).

**Fig. 2:**
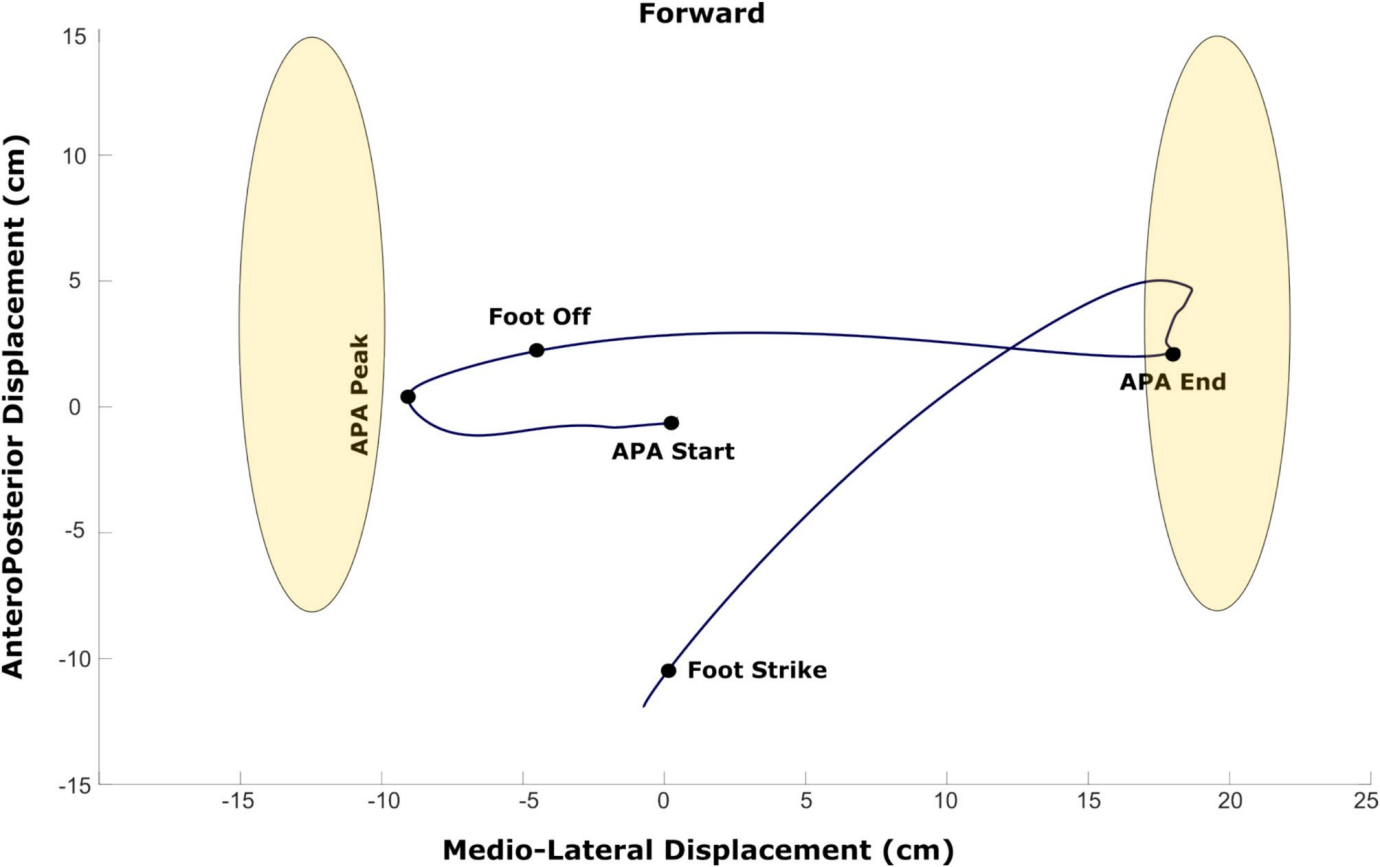
Illustrates the CoP trajectory during the initiation of a counter-clockwise turn from a randomly selected older adult. The CoP trajectory is shown during a left-foot-initiated turn (stepping leg). The light-yellow shapes represent the feet as identified by motion capture markers, while the dark blue line represents the CoP trajectory over time. Black circles highlight the landmarks used to identify and segment the APA phases.

The following landmarks were identified on the CoP trajectories for each time window^8^: i) APA Start: the initial point when the CoP trajectory exceeded the threshold value. ii) APA Peak: the first postero-lateral peak following the APA Start. iii) APA End: the time at which the CoP trajectory shifted from the mediolateral to the anteroposterior direction. APAs features and first step characteristics were extracted as described in (Table 1). Figure 2 provides a graphical summary of the CoP trajectory during the APAs, with key landmarks highlighted.

**Table 1:** Summarises the outcome variables extracted in the study and provides a brief description of how each was estimated.

Variable	Description
APA1 duration (ms)	Time between APA Peak and APA start
APA1 amplitude (AP, ML and total) (cm)	Spatial difference in CoP coordinates between APA peak and APA Start
APA1 velocity (m/s)	APA1 Amplitude Total divided by APA1 duration
APA2 duration (ms)	Time between Foot Off time and APA peak
APA2 amplitude (AP, ML and total) (cm)	Spatial difference in CoP coordinates between foot off and APA Peak
APA2 velocity (m/s)	APA2 amplitude total divided by APA2 Duration
Weight shift duration (ms)	Time between APA peak and APA end
Weight shift amplitude (total) (cm)	Spatial difference in CoP coordinates between APA end and APA peak
Weight shift velocity (m/s)	Weight shift amplitude divided by weight shift duration
Foot off time	First foot motion capture marker of the stepping foot (i.e. toe or heel) to exceed the threshold value
Time to foot off (%)	Percentage ratio of the weight shift phase duration to the total time until foot off
Foot contact	Last foot motion capture marker of the step leg (i.e. either toe or heel) to return under the threshold value
Step length (cm)	Linear distance between the ankle marker positions of the stepping leg, calculated between foot off and foot contact
Step velocity (m/s)	Step length divided by step duration (foot contact – foot off)
BOS (cm)	Linear distance between the ankle markers, measured prior to step initiation

AP = anteroposterior; ML = mediolateral; total = linear distance calculated as $\sqrt{{AP}^{2}+{ML}^{2}}$ ; BOS = base of support.

Spatiotemporal parameters of APAs (i.e., duration, amplitude and velocity) were measured for APA1 (imbalance), APA2 (unloading), and total weight-shift along the mediolateral and anteroposterior components. The linear distance covered by the CoP during a given phase was calculated as the square root of the sum of the anteroposterior and mediolateral components squared. As the APAs amplitude APA1, APA2 and weight-shift are influenced by the BoS^5^ these variables were normalised for the participants’ BoS.

Additionally, CoP trajectories related to the weight-shift were time normalised (500 points) to allow waveform correlation analysis. The spatiotemporal characteristics of the first step were also extracted. During the analysis of the CoP trajectories, signals were visually inspected to identify possible features of APAs in turning that are not typical to APAs in gait initiation.

Video recordings of the trial were used to identify the overall frequencies of turning strategies adopted by participants (i.e., which limb was leading in the context of the turn direction), regardless of whether they stopped or experienced freezing. Additionally, the recordings were used to identify turns that had to be discarded from the analysis due to experimenter’s interventions (e.g., supporting people to prevent falls) or freezing of gait. Specifically, freezing of gait was assessed through the annotation of video recordings by a panel (YR, WY, ZW)^27^. As evidence shows that freezing of gait is often accompanied and/or preceded by a breakdown of the normal APAs pattern, with multiple small, ineffective or absent weight shifts rather than a single organized APAs^29,30^, turns that involved or were preceded by freezing (< 3 s) were excluded from the analysis. We also excluded from the final analysis participants with fewer than three valid turns. Three randomly selected turns per participant were included in the final analysis.

MDS-UPDRS-III scores were used to identify the most and least affected sides in pwP. Only individuals who exhibited asymmetry in motor symptoms (defined as a difference of ≥ 1 point between sides) were included in analyses comparing the most and least affected sides.

### Statistical analysis

APAs, step characteristics and balance scores were summarised using the median and interquartile range. Participants were classified into two balance groups based on Mini-BESTest scores (> 20 = good balance; ≤ 20 = low balance) to explore whether balance ability influenced preference for a given turning strategy. Normality was checked using the Shapiro–Wilk test. Nonparametric tests were used, where appropriate, due to the small sample size. Effect sizes were quantified using the r coefficient (see^31^).

Two-tailed Mann–Whitney U tests were conducted to compare APAs, step characteristics and balance scores, between the two groups, while two-tailed Wilcoxon signed-rank tests were used to compare APAs and first step characteristics in the PD group when turning was initiated with the most affected side versus the least affected side. Spearman’s rank correlation coefficients were calculated to describe the relationships between APAs (total displacement features) and first step characteristics in turning, and between people’s balance and adopted turning strategies (see Table 2).

**Table 2:** Summarises the participants characteristics.

Characteristics	Healthy older adults	People with PD
Sex Female, n (%)	9F (53%)	10F (46%)
Age years, mean ± SD	73.41 ± 8.02	68.96 ± 8.62
Height metres, mean ± SD	1.69 ± .12	1.70 ± .09
Body mass kilograms, mean ± SD	74.84 ± 16.49	75.20 ± 16.94
MiniBEST score, median ± IQR	27.0 ± 3.0	22.0 ± 4.5
MoCA score, mean ± SD	26.1 ± 2.9	27.7 ± 1.9
UPDRS-III score, mean ± SD	**-**	31.68 ± 13.74
H&Y stage, median ± IQR	**-**	2 ± 1
FOG pwPD and FOG, n (%)	**-**	7 (32%)
NFOG score, mean ± SD (only for pwP and FOG)	**-**	22.7 ± 4.46 (7 People)

PD = Parkinson’s disease; MiniBest = mini balance evaluation systems test; MOCA = montreal cognitive assessment; MDS-UPDRS – III = movement disorders society unified Parkinson’s disease rating scale-part III; H&Y = Hoehn and Yahr stage; SD = standard deviation; IQR = interquartile range; FOG = freezing of gait; NFOG = new freezing of gait questionnaire.

Time normalised weight-shift waveforms for each group were compared using the coefficients of multiple correlation (CMC,^32^) to investigate intra-group variability. CMC is commonly used in the context of motion analysis and motor coordination to evaluate the similarity/consistency of waveforms within and/or between groups. It provides a single interpretable metric that reflects how well waveforms align in shape, even if they vary in magnitude.

Statistical analysis was carried out using SPSS (v.28, IBM Corp, Armonk, NY, USA). A customised function written in Matlab was used to perform the CMC analysis. For all analyses, the level of significance was set to α = 0.05.

## Results

### Participants

A total of 22 pwP and 17 older adults were included in the final analysis. Five participants (2 OA, 3 pwP) were excluded due to technical issues during data collection, and sixteen (5 OA, 11 pwP) were excluded because they did not have at least three trials suitable for APA analysis. Excluded trials involved either insufficient quiet stance prior to movement, freezing episodes or experimenters’ interventions. Of 22 pwP included, 7 self-identified as experiencing freezing of gait based on the first item of the NFOG-Q. Two of these experienced freezing during the testing session. Two pwP did not show any asymmetry in motor symptoms (based on MDS-UPDRS-III scores) and therefore were not included in the descriptive analysis of turning strategies. Participant characteristics are summarised in (Table 2).

### Characteristics of APAs in turning

Descriptive data of the APAs and first step characteristics for the older adults’ group are reported in (Table 3). The variability of APAs in turning was high as shown by the reported interquartile ranges and the CMC analysis (see Table 3). In older adults, step length and velocity positively correlated with APA1 (r(15) = 0.627, p = 0.002 and r(15) = 0.534, p = 0.027, respectively), APA2 (r(15) = 0.529, p = 0.029 for step length), and weight-shift (r(15) = 0.574, p = 0.015, and r(15) = 0.490, p = 0.046, respectively).

**Table 3:** Summarises the study’s main results.

Outcome variable	Older adults	People with PD	P value
MiniBest	27.0 ± 3.0	22.0 ± 4.5	** < .001**
BOS (cm)	23.0 ± 6.0	24.0 ± 5.0	.769
APA1 duration (ms)	127 ± 122	130 ± 61	.664
APA1 amplitude AP (%)	0.5 ± 1.0	0.5 ± 0.0	.232
APA1 amplitude ML (%)	0.7 ± 2.0	0.6 ± 1.0	.190
APA1 amplitude total (cm)	1.1 ± 3.0	1.0 ± 1.0	-
APA1 amplitude total (%)	5.0 ± 10.0	5.0 ± 5.0	.181
APA1 velocity (m/s)	0.10 ± .09	0.05 ± .05	** < .001**
APA2 duration (ms)	73 ± 27	64 ± 56	.267
APA2 amplitude AP (%)	0.9 ± 2.0	0.6 ± 1.0	**.008**
APA2 amplitude ML (%)	0.4 ± .0	0.2 ± .0	**.017**
APA2 amplitude total (cm)	1.1 ± 1.0	0.7 ± 1.0	-
APA2 amplitude total (%)	5.0 ± 5.0	2.5 ± 3.0	**.002**
APA2 velocity (m/s)	0.27 ± .49	0.19 ± .30	.457
Weight shift duration (ms)	321 ± 216	328 ± 122	.944
Weight shift amplitude (cm)	10.0 ± 5.0	9.0 ± 2.0	-
Weight shift amplitude (%)	44.0 ± 12.0	37.5 ± 9.0	**.017**
Weight shift velocity (m/s)	0.32 ± .15	0.27 ± .10	.077
Time to foot off (%)	25 ± 10	21 ± 15	.424
Step length (cm)	16.0 ± 17.0	7.5 ± 6.0	**.021**
Step velocity (m/s)	3.01 ± 2.64	1.51 ± 1.14	**.039**
CMC coefficient	0.22	0.18	-

Data is reported as median ± interquartile range. BOS = base of support; m = metres; s = seconds; ms = milliseconds; cm = centimetres; CMC = coefficients of multiple correlation. Significant differences are highlighted in bold. APA1 amplitude total (cm), APA2 amplitude total (cm) and weight shift amplitude (cm) represent the un-normalised amplitudes of the different APA phases.

Older adults showed a clear preference for initiating a turn towards the right (~ 84% of turns). Furthermore, as participants could self-select both the turn direction and the initiating leg, two possible strategies were identified: i) enlarge the BoS by initiating the turn with the leg in the same direction of turn (here called ipsilateral initiation); ii) reducing the BoS by initiating the turn with the leg opposite of the direction of the turn (here called contralateral initiation). No associations were found between balance scores and the adopted turning strategies (p > 0.05). Frequencies of the different turning strategies are reported in (Table 4).

**Table 4:** Summarises the frequency of turns for the Parkinson’s group and the healthy control group, categorized by turning direction and stepping leg. Ipsilateral (CCW/L and CW/R) and contralateral (CCW/R and CW/L) initiations are here presented in detail**.**

	CCW	CW	L	R	CCW/L	CCW/R	CCW R/L	CW/L	CW/R	CW R/L	Total turns
PwP	68	252	140	181	40	28		100	152		320
Percentage	21.25	78.75	43.75	56.56	12.50	8.75	41.18	31.25	47.50	60.32	
OA	39	207	118	128	27	12		91	116		246
Percentage	15.85	84.15	47.97	52.03	10.98	4.88	30.77	36.99	47.15	56.04	

PwP = People with Parkinson’s; CCW = counterclockwise turn; CW = Clockwise turn; L = left stepping foot; R = right stepping foot.

### Comparisons between older adults and PD

Differences between pwP and older adults were found for both APAs and first step characteristics. Specifically, compared to older people, pwP showed smaller APA2 values for anteroposterior, mediolateral and total displacement (U(39) = 94.0, p = 0.008 r = 0.42; U(39) = 103.0, p = 0.017 r = 0.38; U(39) = 81.5, p = 0.002, r = 0.50, respectively) as well as reduced total weight-shifts (U(39) = 103.5, p = 0.017, r = 0.39). PwP showed slower APA1 (U(39) = 76.0 p < 0.001, r = 0.94) but similar velocities of APA2 (U(39) = 160.0 p = 0.457, r = 0.12) and weight-shift (U(39) = 124.0 p = 0.077, r = 0.29) in comparison to older people. Step length was shorter in pwP compared to older people (U(39) = 106.0 p = 0.021, r = 0.37); further, steps were slower in pwP (U(39) = 114.0 p = 0.039, r = 0.33). PwP showed similar variability in APAs compared to older adults (see Table 3). In pwP, there was a lack of significant correlations between step length and velocity with APA1 (r(37) = 0.211, p > 0.05, and r(37) = 0.173, p > 0.05, respectively) and APA2 (r(37) = 0.213, p > 0.05 and r(37) = 0.218 p > 0.05, respectively). Correlations coefficients were similar between older adults and pwP for weight-shift (r(37) = 0.500 p < 0.001 and r(37) = 0.554 p < 0.001, respectively). No other notable differences were observed.

Similarly to older adults, pwP showed a clear preference for initiating a turn towards the right (~ 79% of turns; see Table 4). A significant association was found between balance scores and the frequency of contralateral initiation of turns (r(20) = 0.533 p = 0.016).

### Comparison of APAs between turns towards most/least affected side

People with PD were overall more likely to initiate a turn using strategies that increased their BoS (ipsilateral initiation: ~ 63% of turns) compared to strategies that reduced it (contralateral initiation: ~ 37% of turns, see Table 4). However, pwP with poorer balance (miniBEST < 20) used contralateral initiations less often (13% of turns) than pwP who had better balance (miniBEST ≥ 20, 38% of turns). No clear preferences in turning direction appeared to be dictated by the most/least affected side (see Table 5).

**Table 5:** Summarises the turning strategies of people with PD based on their most/least affected side (according to the MDS-UPDRS-III scores). The asymmetry scores were derived by subtracting the sum of MDS-UPDRS III item scores for the least affected side from the sum for the most affected side. The items included in this calculation were 3.3, 3.4, 3.5, 3.6, 3.7, 3.8, 3.15, 3.16, and 3.17.

Most affected side	Participant	Asymmetry score (M-L)	Turn direction M	Turn direction L	Step M	Step L	Turn M/Step M	Turn M/Step L	Turn L/Step M	Turn L/Step L	Total turns
L	PFOG07	9	5	3	7	1	5	0	2	1	8
L	PFOG08	1	0	10	2	8	0	0	2	8	10
L	PFOG16	6	11	1	10	2	10	1	0	1	12
R	PFOG17	1	13	0	10	3	10	3	0	0	13
L	PFOG18	5	5	4	1	8	0	5	1	3	9
L	PFOG20	5	0	9	2	7	0	0	2	7	9
L	PFOG22	1	0	15	2	13	0	0	2	13	15
L	PNFOG04	10	7	9	4	12	1	6	3	6	16
R	PNFOG05	7	14	9	15	9	12	1	1	8	23
L	PNFOG06	3	1	16	8	9	1	0	7	9	17
R	PNFOG07	4	6	6	3	9	1	5	2	4	12
R	PNFOG08	1	12	1	5	8	5	7	0	1	13
R	PNFOG09	5	8	5	9	4	6	2	3	2	13
L	PNFOG10	4	8	9	6	11	4	4	2	7	17
L	PNFOG11	5	0	14	0	14	0	0	0	14	14
R	PNFOG13	7	11	2	8	5	8	3	0	2	13
L	PNFOG14	3	0	16	15	1	0	0	15	1	16
R	PNFOG17	8	17	0	8	9	8	9	0	0	17
L	PNFOG20	5	5	10	2	13	0	5	2	8	15
R	PNFOG21	6	12	3	7	8	6	6	1	2	15
Percentage			48.74	51.26	44.60	55.40	27.90	20.65	16.30	35.15	13.85

R = right; L = left; M = most affected side; L = least affected side.

Since most participants demonstrated a clear preference for a specific turning direction (see Table 4), only 8 participants with PD (with motor symptom asymmetry and with at least three viable trials in both directions) could be included in the comparison of APAs and step characteristics between the most and least affected sides. No significant differences were observed (p > 0.05) in either APAs or step characteristics between turns towards the most versus least affected side.

## Discussion

The current study characterised APAs prior to turning in older adults and pwP. Our findings show that APAs in turning share several features with those preceding forward gait initiation ^4,7,8^, albeit with preparatory adjustments primarily occurring along the mediolateral direction. Additionally, our results highlight key differences between older adults and pwP in both APA features (i.e., amplitude but not duration) and first-step characteristics (i.e., step length and velocity). Interestingly, significant associations between APA features and step execution were found only in older adults, suggesting that PD may disrupt the relation between task preparation and execution during turning.

### APAs in turning: features in semi-prescribed tasks

APAs prior to voluntary turning follow a similar fundamental pattern to those observed in forward gait initiation^4,7^, reflecting the postural preparation required to displace the body during bipedal movements. Before turning, the CoP shifts backward and laterally under the stepping foot, transferring body weight onto the standing limb. This shift ensures a safe foot-off by reducing the distance between the centre of mass and the medial margin of the BoS. However, compared to forward gait initiation^7,8^, and in line with our hypothesis, our data show that APA1 amplitude tends to be smaller and shorter during turning, aligning more closely with APA1 reported in lateral stepping^19^. We attribute this difference to the fact that, while turning on the spot does not require the anterior acceleration needed in forward gait initiation, it still demands a small and precise forward shift to avoid instability.

APA2 and weight-shift characteristics did not show distinguishable differences from forward gait initiation, likely because these components are primarily related to the effective transfer of body weight onto the standing leg. However, we observed greater variability in these components during turning, as indicated by higher interquartile ranges and a much lower CMC. For example, in forward gait initiation, weight shift typically exhibits very low inter-personal variability (CMC = 0.90)^8^, while in the current study, we found substantial variability (CMC = 0.22). Although some of this difference could be attributed to the more controlled nature of the task performed in Russo & Vannozzi’s study and the higher number of trials, we believe that the increased variability observed in this study is more likely produced by the greater complexity of turning. In fact, turning involves movements along the transverse plane and allows for more strategies (i.e., turn direction and stepping leg) compared to forward gait initiation, which is limited to selecting the stepping leg.

Another aspect that could have influenced the observed variability was the design of the turning task. While certain aspects were deliberately constrained (e.g., all turns were performed on the spot) to ensure comparability across trials and participants, we also allowed participants to select which direction they would turn first and which stepping foot to use to introduce a degree of choice intended to reflect more daily-life turning characteristics. This approach, however, may have introduced variability not necessarily reflective of each individual’s typical daily-life preferences. Such semi-prescribed choices likely enhanced ecological relevance by permitting natural variation in strategy, but at the same time may have increased between-trial and between-person variability compared to forward gait initiation, where fewer degrees of freedom exist and behaviour is more stereotyped. Thus, we suggest that the variability we observed in this study likely reflects the combined influence of the intrinsic complexity of turning and the additional behavioural flexibility afforded by our task design, and as such may not be necessarily transferable to real-life scenarios where turning does not always occur on the spot and may be influenced by other factors such as environmental demands (e.g. furniture, goal-directed actions), or emotional and physical state. Indeed, future studies should further investigate the variability of APAs in real-life situations.

### APAs in turning: effects of Parkinson’s disease

In line with our initial hypothesis, individuals with PD demonstrated impoverished APAs compared to older adults. Specifically, pwP exhibited reduced APA2 amplitude and smaller weight-shift components, as well as slower execution of APA1. These findings are consistent with previous research on forward gait initiation, where impaired APAs in PD were linked to difficulties in task execution and balance control^5,10,12^. The smaller APAs amplitudes observed in pwP (APA2 and weight-shift) and the slower APA1 suggest a reduced capacity to adequately prepare for the dynamic demands of turning, potentially increasing the risk of falls during everyday tasks involving changes in body orientation due to altered weight shifts^33^. Furthermore, this may be indicative of deficits in both motor and postural control, suggesting an altered ability to precisely adjust the spatiotemporal characteristics of APAs to align with the motor program for the turning step, which may further compromise stability during turning. Taken together, these results may explain why pwP were overall more likely to adopt strategies that increased their BoS when initiating turning (~ 63% of turns), compared to strategies that reduced it (~ 37%). This preference for stability-enhancing strategies may reflect an adaptive response to their postural control impairments, as expanding the BoS may provide greater stability during dynamic movements such as turning.

Interestingly, while pwP exhibited shorter and slower steps during turning initiation, no significant differences were found between the two groups for foot-off percentage or the duration of any of the APA components. Several explanations may account for these observations. First, the combination of shorter and slower steps in pwP compared to OA may suggest a less effective turning strategy, in which step length is limited as a compensatory mechanism to preserve stability, albeit at the expense of efficiency. This interpretation is consistent with the observation that spatial, but not temporal, aspects of APAs differed between groups, and may also explain why no associations between APAs and step characteristics were identified in pwP. Alternatively, it is possible that the lack of difference in the temporal characteristics of APAs may be partly confounded by our inclusion criteria (e.g., balance abilities) and the relatively small sample size (when taking into account the overall variability observed). Lastly, we speculate that our results could indicate that the temporal components of APAs in turning might be less impaired in the early stages of PD, especially during semi-prescribed turning, where preparatory mechanisms may be more preserved compared to spatial ones.

An important aspect of our study was the relatively high proportion of excluded turns, particularly in the pwP group. While exclusions were based on strict methodological criteria (i.e., ensuring sufficient quiet stance before APA onset), it is possible that some of the excluded turns were initiated with strategies that did not display the characteristic APA components (APA1, APA2, weight-shift). The potential absence of specific APA components would not be unique to turning; indeed, previous work has shown that during forward gait initiation some individuals adopt strategies in which one or more APA components are missing^9^. Future research using more prescribed/regimented protocols should examine the frequency and distribution of these different APA patterns in greater detail, as their presence or absence may provide valuable insight into how individuals adapt anticipatory control mechanisms, and how these adaptations may be affected in populations with balance impairments.

### Turning side and APA characteristics

Our analysis of turns towards the most versus least affected side in pwP did not reveal significant differences in APAs or step characteristics. Furthermore, pwP overall preferred to use turning strategies that prioritised the increase of the BoS rather than showing a preference based on most/least affected side, which may indicate that, different to forward gait initiation, asymmetries in disease severity play only a minor role during turning^34^. Although we admittedly only had a relatively small sample size (8 pwP), these findings corroborate results recently published by Seuthe and co-workers^34^ who identified asymmetries in gait but not turning in people with mild-to-moderate PD. A possible explanation is that asymmetry may be apparent in relatively simple task (e.g., gait initiation), but it may be masked/no longer apparent in more complex tasks where other aspects (e.g., turning strategy) may play a more pivotal role. Turning requires a fine coordination between body segments and, compared to forward gait, it poses greater challenges for sensory integration^35^ and requires greater cognitive demands^36^. Furthermore, these factors are likely to be further exacerbated in less prescribed/contrived adaptive gait tasks, such as that used in the current study. Therefore, while this more naturalistic behaviour could serve to mask subtle differences, the current data suggests that these differences are not of a sufficient magnitude to be clinically important.

### Potential implications for rehabilitation and clinical practice

Research indicates that turning is more vulnerable to balance and functional impairments than forward walking due to its complex demands^37,38^. In fact, during turning, pwP are more likely to experience freezing of gait or falls^16,22^. Impairments in APAs are considered a major contributor to difficulties with gait initiation in pwP^15^, and dysfunctional APAs have been proposed as a possible cause of freezing of gait^39^. However, it is often challenging to observe differences in APAs during gait initiation in participants who are ON-medication, as dopamine tends to improve APAs^5^ especially in people with moderate PD and without freezing of gait^17^, potentially masking PD-related impairments in balance control. However, despite our relatively small sample consisting of individuals with mild-to-moderate PD tested ON medication, we were able to detect significant differences both in the preparatory phase of turning and in the first step. Although more work is necessary to improve the generalisability of our results, the sensitivity of turn initiation in detecting subtle changes in postural control makes it a promising candidate for its inclusion in clinical assessments.

While turning is generally associated with a narrower BoS in pwP^40^, we did not observe differences in self-selected BoS prior to turns in this semi-prescribed task. However, our results highlighted a significant association between balance ability and the adopted turning strategy in pwP, with higher balance scores linked to more frequent use of contralateral turn initiation, implying a reduced BoS. Interestingly, this relationship was not observed in older adults. Reducing the BoS when initiating a turn may represent a more challenging strategy as there is potential instability at the end of the first step, due to momentum in the direction of the turn that could push the centre of mass beyond the margins of the BoS. We believe that, for individuals with good balance, contralateral initiation does not pose a significant threat. As such, we would not expect to observe any relationship between balance ability and turn-initiation strategy in older adults. In contrast, pwP who demonstrated poorer overall balance, may adopt the ipsilateral initiation strategy as an active avoidance mechanism to reduce postural instability during turning.

Overall, pwP with poorer balance (miniBEST < 20) initiated turns using a contralateral strategy 13% of times, while those with better balance (miniBEST ≥ 20) did so 38% of times. This suggests that people still tend to adopt a strategy, when under no specific time-pressure, that poses a non-negligible risk to balance. These findings are reminiscent of observations of behavioural risk-taking in older adults^41,42^, and the use of cross-stepping in nursing homes residents^43^. In these studies, participants either selected higher risks tasks^41,42^ or unnecessarily opted for strategies (i.e., cross-stepping) which narrow the BoS, thus posing an increased challenge for the balance system. In the context of this study, cross-stepping may be viewed as an extreme example of contralateral turn initiation. As such, this type of behaviour could be maladaptive and enhance fall risk.

Furthermore, as BoS reduction during turning is thought to be a key factor in falls^40^, this suggests a possible rehabilitation strategy: encouraging pwP who have high instability to avoid transitioning directly from walking to turning, but instead to break the task into two distinct steps. Recent evidence suggests that especially pwP who experience freezing could benefit by incorporating pauses/breaks between tasks or during a freeze to regain control over their balance^44^.

### Limitations and future directions

This study has three main limitations. i) Semi-prescribed turning task resulting in a significant loss of data suitable for APA analysis (e.g., absence of pauses or lack of turns in a given direction among pwP). However, we believe this is also a key strength of the study, as it provides valuable insights into turning initiation in a naturalistic context, making the findings more applicable to real-life situations. ii) Small sample size for comparing turns between the most and least affected sides in PD. The small sample size limited our ability to fully assess side-specific impairments in turning initiation. Future research with larger samples is needed to better understand how such asymmetries impact turning initiation in pwP. Additionally, although participants were assessed in their ON-medication state, the specific effects of medication on APAs characteristics during turning were not explored, which represents an important area for future investigation. iii) Exclusion of participants with poor balance. Since individuals with poor balance, who are typically in the later stages of PD, were excluded from this study, the generalisability of these findings is limited. Including participants with more advanced balance impairments in future research would provide a more comprehensive understanding of turning difficulties across the full spectrum of PD severity.

## Conclusion

In summary, APAs prior to turning share several similarities with APAs prior to gait initiation. When compared to older adults, people with PD exhibit significant impairments in APAs execution during turning, including reduced amplitude and slower execution, which may contribute to the high incidence of falls during turning in this population. Our findings underscore the need for targeted interventions to address these specific motor deficits and suggest that strategies promoting weight-shifting and a stable base of support may aid in mitigating fall risk. Future research should continue to explore the interplay between motor asymmetry, medication effects, and turning performance to further refine clinical approaches to balance rehabilitation in PD.

## Data Availability

The data that support the findings of this study are available from the corresponding author upon reasonable request.
